# Behavioral risk factors of breast cancer in Bangui of Central African Republic: A retrospective case-control study

**DOI:** 10.1371/journal.pone.0171154

**Published:** 2017-02-08

**Authors:** Augustin Balekouzou, Ping Yin, Henok Kessete Afewerky, Cavin Bekolo, Christian Maucler Pamatika, Sylvain Wilfrid Nambei, Marceline Djeintote, Antoine Doui Doumgba, Christian Diamont Mossoro-Kpinde, Chang Shu, Minghui Yin, Zhen Fu, Tingting Qing, Mingming Yan, Jianyuan Zhang, Shaojun Chen, Hongyu Li, Zhongyu Xu, Boniface Koffi

**Affiliations:** 1 Department of Epidemiology and Biostatistics, School of Public Health, Tongji Medical College of Huazhong University of Sciences and Technology, Wuhan, China; 2 Department of Pathology and Pathophysiology, Tongji Medical College of Huazhong University of Sciences and Technology, Wuhan, China; 3 Ministry of Public Health, Centre Medical d’Arrondissement de Bare, Nkongsamba, Yaoundé, Cameroon; 4 Hospital Laboratory Friendship of Bangui, Bangui, Central African Republic; 5 Faculty of Health Sciences, University of Bangui, Bangui, Central African Republic; 6 National Laboratory of Clinical Biology and Public Health, Bangui, Central African Republic; University of Oklahoma Health Sciences Center, UNITED STATES

## Abstract

Breast cancer is recognized as a major public health problem in developing countries; however, there is very little evidence of behavioral factors associated with breast cancer risk. This study was conducted to identify lifestyles as risk factors for breast cancer among Central African women. A case-control study was conducted with 174 cases confirmed histologically by the pathology unit of the National Laboratory and 348 age-matched controls. Data collection tools included a questionnaire with interviews and medical records of patients. Data were analyzed using SPSS software version 20. Odd ratio (OR) and 95% confidence intervals (95% CI) were obtained by unconditional logistic regression. In total, 522 women were studied with a mean age of 45.8 (SD = 13.4) years. By unconditional logistic regression model, women with breast cancer were more likely to have attained illiterate and elementary education level [11.23 (95% CI, 4.65–27.14) and 2.40 (95% CI, 1.15–4.99)], married [2.09 (95% CI, 1.18–3.71)], positive family history [2.31 (95% CI, 1.36–3.91)], radiation exposure [8.21 (95% CI, 5.04–13.38)], consumption charcuterie [10.82 (95% CI, 2.39–48.90)], fresh fish consumption [4.26 (95% CI, 1.56–11.65)], groundnut consumption [6.46 (95% CI, 2.57–16.27)], soybean consumption [16.74 (95% CI, 8.03–39.84)], alcohol [2.53 (95% CI, 1.39–4.60)], habit of keeping money in bras[3.57 (95% CI, 2.24–5.69)], overweight [5.36 (95% CI, 4.46–24.57)] and obesity [3.11(95% CI, 2.39–20.42)]. However, decreased risk of breast cancer was associated with being employed [0.32 (95% CI, 0.19–0.56)], urban residence [0.16 (95% CI, 0.07–0.37)], groundnut oil consumption [0.05 (95% CI, 0.02–0.14)], wine consumption [0.16 (95% CI, 0.09–0.26)], non habit of keeping cell phone in bras [0.56 (95% CI, 0.35–0.89)] and physical activity [0.71(95% CI, 0.14–0.84)]. The study showed that little or no education, marriage, positive family history of cancer, radiation exposure, charcuterie, fresh fish, groundnut, soybean, alcohol, habit of keeping money in bras, overweight and obesity were associated with breast cancer risk among Central African women living in Bangui. Women living in Bangui should be more cautious on the behavioral risk associated with breast cancer.

## Introduction

Breast cancer (BC) is one of the commonly diagnosed cancers that leads to mortality and morbidity in women worldwide [1]. Nowadays, it is the leading cause of cancer death in women with 198,000 deaths per year, representing 15.4% of deaths in developed regions after that of lung [2]. Furthermore, in developing countries, it is the first leading cause of death among women with 324,000 deaths which represents for 14.3% of all deaths [3]. Moreover, in 2012, this rate varied between 6 and 20 per 100,000 in East Asia and West Africa [4].

In addition, the number of new cases diagnosed in women was 1.7 million (25% of all cancers), with 883,000 cases observed in developed regions against 794,000 cases in developing countries [4,5]. Incidence rates vary almost from single to quadruple from one region to another throughout the world [4]. In 2012, the incidence rate in Africa and East Asia was 27 per 100,000, while in Europe it was 96 per 100,000 [4]. In 2011, the annual incidence rate in Sub-Saharan Africa (SSA) was 22 per 100,000 women [6]. The age distribution of the incidence of BC can objectify a high incidence increasing from 35 years, peaking at 60 [7].

According to the 2012 report by the World Health Organization (WHO), breast cancer-related mortality is the leading cause of death (24.5%) among Central African women [8,9]. The recent reports by the International Agency for Research on Cancer (IARC) suggest that global trends in developing countries are rapidly progressing according to social changes, economic and lifestyle, compatible with industrialized countries, which leads to increase burden of cancers associated with risk factors including hormonal or nutritional [4]. In addition, some behavioral risk factors, such as traditional lifestyles are often associated with an increased risk of BC [10]. Therefore, Mc Cormack and Buftak estimates the global burden of cancer between 13.3 million and 21.4 million in countries with lower income to moderate during 2010–2030 according changing demographics. Risk factors associated with lifestyle change in these countries will have an impact on the increasing prevalence [11].

Several studies have reported independent effects of various lifestyle factors such as eating habits, physical activity, smoking, alcohol consumption and anthropometric characteristics, such as Body Mass Index (BMI) with the incidence of BC [10], sedentary lifestyle, with a bad diet regime, especially during the last 20 years was associated with a risk of obesity and weight gain [12]. Physical activity is associated with a reduced risk of BC by affecting weight gain and obesity, insulin resistance and chronic inflammation [13]. According to the results of the study carried out by Mayor S. in Italy (2011) on the effect of changes in lifestyle to reduce the risk of BC, abstinence from alcohol, the practice of physical activity (minimum 2 hours per week), and BMI less than 25 Kg/m^2^, will significantly reduce the risk of BC [14]. Therefore, women should be educated to change their lifestyles to reduce the risk of BC.

In Central African Republic (CAR), diseases induced by lifestyle are considered to be the main causes of disability and death. In addition, in recent decades, when the CAR began recording a significant reduction of infectious diseases through different national programs implemented, new diseases, including cancer and other non-communicable chronic diseases began emerging as new public health priorities. Unfortunately, only few hospital studies had been conducted in this domain, and none have studied the risk factors associated with this disease in the population [15,16].

Given increasing prevalence of BC in CAR, the absence of an operational plan for the fight against cancer and the lack of appropriate support structure, it is necessary to study the lifestyle factors and risk in people with this disease. Thus, this case-control study aimed to identify lifestyle factors associated with BC risk in women with BC confirmed by Pathology unit of the National Laboratory of Bangui. The data obtained will allow for the development of new strategies to fight this disease and its complications.

## Materials and methods

This case control study was carried out in the pathology unit of the National Laboratory, and the general surgery and gynecology services of two tertiary care institutions in Bangui, CAR.

### Study population

The studied population was women, included in the cases and controls. All cases were women with invasive BC histologically confirmed between September 2003 and September 2015. The controls were randomly recruited from women who came for other pathologies at the National Laboratory of Bangui. For each case, two controls were included in the study. All controls were free of any cancer. They were matched for age (same age) because BC is an age-related disease and increasing age is the single most important risk factor after female gender [17]. In addition, all controls were considered to come from the same urban community as the cases. The patients were ethnically and socioeconomically diverse and represented the diversity of the Central African population and the patient population that comes from the general surgery and gynecology services that are separate institutions, even if they are located in the same city of Bangui, capital of the CAR.

### Inclusion and exclusion criteria

All women living in Bangui, aged ≥ 15 years, who presented with BC, which was confirmed by histology, and gave consent were included in the cohort/study. However, all women aged less than 15 years, not residing in Bangui or those whose pathological diagnosis dates were outside the study period or refused to participate in the study were excluded.

### Recruitment of participants

Investigators reviewed the records and reports of laboratory for each patient to identify BC cases confirmed during the study period. Information about each patient was recorded on a data collection sheet. Then the contact was made with the consultant physicians to supplement information on certain medical parameters and addresses of patients. For cases of those who had died, relatives were selected as next of kin to provide data relating to the patient’s lifestyle. The controls were randomly recruited from women who came for other pathologies at the National Laboratory of Bangui.

### Data collection

Data was collected from a cancer register held in the pathology unit of the National Laboratory; from medical records of patients seen at the general surgery and gynecology services. The main details of the risks and benefits of the study were explained to all eligible participants. Those who agreed signed an informed consent form before the interview. This interview was conducted in Sango (second official language in CAR). For participants who did not understand Sango, adult relatives interpreted the content of the questionnaire and consent form for better understanding. For minors or children, the written consent form was obtained from close relatives or caretakers before being enrolled in the study. Each potential participant had the choice to accept or refuse to participate in the study. Questions were also granted to volunteers who wish clarification.

### Study variables

The following variables were considered: age, occupation, economic status, education level, areas of residence (urban or rural), ethnic group and marital status. In addition, family history of cancer was included as hereditary factors. Finally, radiation exposure and certain foods consumed in CAR as cassava, rice, charcuterie, red meat, vegetables, fish, groundnuts, palm oil, soybean, coffee, physical activity, alcohol, tobacco, use of bra, habit to keep money or cell phones in bras, height, weight and BMI were included as lifestyle factors.

Age was categorized as age groups. The occupation was classified as housewife (for all women who have no job) or as being employed. Economic status has been defined in terms of family income according to international poverty threshold. Low income if below 2 dollars a day, moderate between 2 and 4 dollars, good between 5 and10 dollars and excellent above 10 dollars [18]. The residence was urban for those living in Bangui and rural for those living in the provinces. Ethnicity has been grouped into six major ethnic groups of the country. The education level was classified into illiterate, elementary, high school and university. Marital status was classified as married and single (included divorced and widow). Charcuterie was defined as a set of all dried meats by fire or sun and included beef, cow, pig and others. The practice of physical activity was defined as any movement requiring physical effort, like to move on foot from one point to another every day.

Measurements of height and weight of the control subjects were taken by the investigators. The height was measured using a vertical strip affixed to the wall and the weight with a calibrated scale. BMI was calculated by dividing weight by height squared (kg / m^2^) and women were grouped into different categories such as recommended by WHO; normal range (BMI ≤ 24 kg / m^2^), overweight (BMI = 25–29.9 kg / m^2^) and BMI ≥ 30 kg / m^2^ is synonymous with obesity [19].

### Statistical analysis

Pearson chi-square (*χ*^2^) tests or Fisher’s exact were used to compare the frequency distribution of categorical variables and whereas *t*-tests Student were used to compare the mean values for the continuous variables between various groups of cases and controls. Unconditional logistic regression models were used to estimate odds ratio (OR) and 95% confidence intervals (CI) for the association between hereditary or lifestyle factors and BC. Based on the comparison of baseline characteristics between cases and controls, the following variables, including occupation, economic status, education level, residence area, marital status, family history of cancer, radiation exposure, cassava flour, rice, charcuterie, red meat, vegetables, fish, groundnuts, palm oil, soybean, coffee, physical activity, alcohol, tobacco, use of bras, habit of keeping money or cell phones in bras, height, weight and BMI were selected to be adjusted for as potential confounding factors. Potential confounders were included in a multivariate logistic regression model when the Chi-square test or *t*-test showed a positive association with BC or if the inclusion of the variable in the analysis changed the risk estimation by ≥10%. All statistical tests were based on a probability of 2 sides, with a significance level of 0.05, and were performed using Statistical Package for Social Sciences (SPSS Inc., Chicago, IL, USA) Version 20.

### Ethical approval and consent to participate

The study was approved by International Review Boards of School of Public Health, Tongji Medical College of Huazhong University of Science and Technology (IRB Approval File No.[2014] 09), and University of Bangui (No 2068/UB/FACSS/CSCVPER/16) according to standards of the Declaration of Helsinki. All participants gave informed written consent.

## Results

In total, 174 cases and 348 controls matched for ages were included. The response rate was 85.99% (522/607). The age at diagnosis for the cases ranged from 16 to 90 years with a mean of 45.83 (SD = 13.5) years. The mean age for the control was 45.79 (SD = 13.3) years (Table 1).

**Table 1:** Socio-demographic characteristic of study participants.

Variables	Cases (174)	Controls (348)	Total (522)	χ2	P	Variables	Cases (174)	Controls (348)	Total (522)	χ2	P
	Freq (%)	Freq (%)	Freq (%)				Freq (%)	Freq (%)	Freq (%)		
**Age group**						**Residence**				21	**0.000**^b^
**15–39**	57 (32.8)	112 (32.2)	169(32.4)			Urban	149 (85.6)	336 (96.6)	485(92.9)		
**40–44**	25 (14.4)	54 (15.5)	79(15.1)			Rural	25 (14.4)	12 (3.4)	37(7.1)		
**45–49**	18 (10.3)	34 (9.8)	52(10.0)			**Ethnic group**				24.88	**0.000**^b^
**50–54**	33 (19.0)	68 (10.5)	101(19.3)			Banda	32 (18.6)	111 (32.2)	143(27.7)		
**55–59**	15 (8.6)	30 (8.6)	45(8.6)			Gbaya	22 (12.8)	67 (19.4)	89(17.2)		
**60–64**	10 (5.7)	18 (5.2)	28(5.4)			Mandja	33 (19.2)	54 (15.7)	87(16.8)		
**65–69**	5 (2.9)	12 (3.4)	17(3.3)			Sara	24 (14.0)	46 (13.3)	70(13.5)		
**70–74**	6 (3.4)	10 (2.9)	16(3.1)			Yakoma	38 (22.1)	48 (13.9)	86(16.6)		
**≥75**	5 (2.9)	10 (2.9)	15(2.9)			Ngbaka	23 (13.3)	19 (5.5)	42(8.2)		
**Mean age (SD)**	45.83 (13.55)	45.79 (13.34)	45.80 (13.40)	t = -0.035	p = 0.97^a^	**Marital status**				18.28	**0.000**^c^
**Occupation**				11.36	**0.001**^b^	Married	42 (24.1)	32 (10.1)	77(14.8)		
**Housewife**	121 (69.5)	287 (82.5)	408(78.2)			Single	132 (75.9)	313 (89.9)	445(85.2)		
**Employed**	53 (30.5)	61 (17.5)	114(21.8)			**Parity**				9.6	**0.008**^c^
**Economic status**				9.92	**0.01**^c^	Nulliparus	31 (17.9)	34 (9.8)	65(12.5)		
**Poor**	24 (13.8)	56 (16.1)	80(15.3)			1–2	46 (26.6)	128 (36.8)	174(33.4)		
**Moderate**	99 (56.9)	231 (66.4)	330(63.2)			≥ 3	96 (55.5)	186 (53.4)	282(54.1)		
**Good**	48 (27.6)	55 (15.8)	103(19.7)								
**Excellent**	3 (1.7)	6 (1.7)	9(1.7)								
**Education level**				22.2	**0.000c**						
**Illiterate**	45 (25.9)	34 (9.8)	79(15.1)								
**Elementary**	57 (32.8)	144 (41.4)	201(38.5)								
**High School**	49 (28.2)	119 (34.2)	168(32.2)								
**University**	23 (13.2)	51 (14.7)	74(14.2)								

Freq = frequency; χ2 = chi-square; SD = standard deviation; P = p-value. χ2 was calculated by using Fisher’s exact chi-square test.

^*a*^ *p*-value was calculated by using *t*-test.

^*b*^ *p*-value was calculated by using Pearson’s chi-square test.

^c^*p*-value was calculated by using Fisher’s exact chi-square test.

Frequency was calculated by using Cross tabulation analyze. Employee includes all sectors: public and private. Poor economic status (income < 2 dollars a day), moderate (income = 3 to 4 dollars a day), good (income = 5 to 10 dollars per day) and excellent (income > 15 dollars a day). Residence: Town (Bangui) and Rural (outside Bangui).

### Socio-demographic characteristics

Table 1 shows the distribution of the characteristics of socio-demographics for the cases and for the controls. Chi-square (χ^2^) tests revealed significant differences between cases and controls with respect to occupation (p = 0.001), economic status (p = 0.01), education level (p < 0.001), area of residence (p <0.001), ethnic group (p< 0.001), marital status (p <0.001) and parity (p = 0.008). There was no significant difference in the mean age observed between BC patient and control group. Over 69% (121/174) of the cases as compared to the controls 82% (287/348) were housewives with a moderate economic status (56.9% and 66.4%). Nearly 13% (23/174) and 14% (51/348) of the cases and controls, respectively, had a level of academic study and lived in cities (85.6% and 96.9%). The most represented ethnic groups were the Banda (18.6% of cases and 32.2% of controls), the Gbaya (12.8% and 19.4%), followed by Mandja (19.2% and 15.7%) and Yakoma (22.1% and 13.9%). Unmarried women held 75.9% (132/174) in the case group against 89.9% (313/348) in the control group, however, a small proportion of cases (17.9%) and controls (9.8%) were nulliparous.

### Socio-demographic factors and their association with breast cancer

The odds for the socio-demographic factors associated with risk of BC were summarized in Table 2. Odds of BC were 11.23 and 2.40 times higher (95% CI, 4.65–27.14; p <0.001and 95% CI, 1.15–4.99; p = 0.01) for women with illiterate or less educated compared to women with a high level of education. For the marital status, the odds of having BC were 2.09 times higher among married women compared to the single (95% CI, 1.18–3.71, p = 0.01). On the other hand, occupation and residence area have a significant protective effect on BC: Women employed and those living in cities showed decreased odds ratio of 0.32 (95% CI, 0.19–0.56; p < 0.001) and 0.16 (95% CI, 0.07–0.37; p < 0.001), respectively. Age at marriage was not a risk associated with BC.

**Table 2:** Socio-demographic factors and their association with breast cancer.

Factors associated	Cases (174)	Controls (348)	Univariate analysis			Multivariate analysis		
	N (%)	N (%)	crude OR	95%, CI [L-U]	P	aOR	95%, CI [L-U]	P
**Occupation**								
**Housewife**	121 (69.5)	287 (82.5)	1.00 (Ref)			1.00 (Ref)		
**Employed**	53 (30.5)	61 (17.5)	**0.31**	[0.31–0.74]	**0.001**	**0.32**	[0.19–0.56]	**0.000**
**Education level**								
**Illiterate**	45 (25.9)	34 (9.8)	**2.93**	[1.51–5.70]	**0.001**	**11.23**	[4.65–27.14]	**0.000**
**Elementary**	57 (32.8)	144 (41.4)	**1.87**	[1.49–4.56]	**0.02**	**2.4**	[1.15–4.99]	**0.01**
**High School**	49 (28.2)	119 (34.2)	0.91	[0.50–1.65]	0.76	1.76	[0.86–3.61]	0.12
**University**	23 (13.2)	51 (14.7)	1.00 (Ref)			1.00 (Ref)		
**Residence**								
**Urban**	149 (85.6)	336 (96.6)	**0.21**	[0.10–0.43]	**0.000**	**0.16**	[0.07–0.37]	**0.000**
**Rural**	25 (14.4)	12 (3.4)	1.00 (Ref)			1.00 (Ref)		
**Marital status**								
**Married**	42 (24.1)	32 (10.1)	**2.84**	[1.73–4.65]	**0.000**	**2.09**	[1.18–3.71]	**0.012**
**Single**	132 (75.9)	313 (89.9)	1.00 (Ref)			1.00 (Ref)		
**Married status by age group**‡								
**< 25**	1 (2.4)	1(3.1)	1.2	[0.46–3.25]	0.66	NA		
**25–39**	12(28.6)	8(25.0)	0.9	[0.65–1.50]	0.98			
**> = 40**	29(69.0)	23(71.9)	1.00 (Ref)					

L = lower; U = upper; crude OR = crude odds ratio; aOR = adjusted odds ratio; Ref = reference; P = p-value; CI = Confidence interval; NA = not applicable. OR was calculated by using logistic regression.

^‡^ This parameter was calculated only for married women.

### Hereditary and lifestyle factors and their association with breast cancer

Predictor effects (risk factors) on BC in Central African women have been shown in Tables 3 and 4. The unconditional logistic regression model identified family history of cancer, radiation exposure, charcuterie consumption, fresh fish, groundnut, soybean, alcohol and habit to keep money in bras as more likely to increase the risk for BC. Indeed, women with a family history of cancer showed 2.31 times higher BC risk (95% CI, 1.36–3.91; p = 0.002), compared to those who have not family history of cancer. However, the odds of having BC were 8.21 times higher among women who had been exposed to radiation before the diagnosis compared to those never exposed (95% CI, 5.04–13.38; p <0.001). Similarly, women who regularly consume charcuterie or fresh fish showed respectively 10.82 and 4.26 times higher BC risk (95% CI, 2.39–48.90, p = 0.002 and 95% CI, 1.56–11.65, p = 0.005) compared to those who do not consume regularly (Table 3). The factors "groundnut consumption", "soybean consumption" and "alcohol" were respectively 6.46; 16.74; 2.53 times higher BC risk compared to those who are not related to these factors. On the other hand, "type of alcohol consumed" was less associated with BC risk (OR = 0.16; 95% CI, 0.09–0.26; p <0.001), especially for women with habit to drink wine compared to those with habit to drink beer. Similarly, regular groundnut oil consumption and no habit of keeping cell phones in bras showed a protective effect against BC (OR = 0.05; 95% CI, 0.02–0.14; p <0.001 and OR = 0.56; 95% CI, 0.35–0.89; p <0.01). In addition, women who practiced physical activities showed a lower odds ratio 0.71 (95% CI, 0.14–0.84; p = 0.01), compared to those not practicing physical activity (Table 4). For women with habit to consume cassava flour (basic foods CAR) and palm oil, odds of having BC were 3.18; 4.50 times higher compared to those with non habit of consuming these foods. Unfortunately, these associations were not statistically significant by unconditional logistic regression (p = 0.46, p = 0.13) (Table 3). Similarly, habit of consuming unsweetened coffee showed a protective effect against BC (OR = 0.05; 95% CI, 0.007–0.36; p = 0.003). But, this association was not statistically significant (OR = 0.20; 95% CI, 0.02–1.72; p = 0.14) (Table 4).

**Table 3:** Univariate and multivariate analysis of breast cancer, history of cancer, radiation exposure and food consumed among women studied.

Factors associated	Cases (174)	Controls (348)	Univariate analysis			Multivariate analysis		
	N (%)	N (%)	Crude OR	95%, CI [L-U]	P	aOR	95%, CI [L-U]	P
**Positive family history of cancer**								
Yes	60(34.5)	56 (16.1)	**2.70**	[1.79–4.19]	**0.000**	**2.31**	[1.36–3.91]	**0.002**
No	114 (65.5)	292 (83.9)	1.00 (Ref)			1.00 (Ref)		
**Breastfeeding by age group**‡								
< 25	3(2.3)	10(3.5)	0.42	[0.05–3.59]	0.43	NA		
25–39	41(31.3)	70(24.2)	0.79	[0.26–2.37]	0.67			
> = 40	87(66.4)	209(72.3)	1.00 (Ref)					
**Radiation exposure**								
Yes	90 (51.7)	41 (11.8)	**8.02**	[5.16–12.47]	**0.000**	**8.21**	[5.04–13.38]	**0.000**
No	84 (48.3)	307 (88.2)	1.00 (Ref)			1.00 (Ref)		
**Regular consumption cassava flour**								
Yes	169 (97.1)	318 (91.4)	**3.18**	[1.21–8.36]	**0.01**	1.73	[0.39–7.63]	0.46
No	5(2.9)	30 (8.6)	1.00 (Ref)			1.00 (Ref)		
**Regular consumption charcuterie**								
Yes	170 (97.7)	245 (70.4)	**17.86**	[6.45–49.44]	**0.000**	**10.82**	[2.39–48.90]	**0.002**
No	4 (2.3)	103 (29.6)	1.00 (Ref)			1.00 (Ref)		
**Regular consumption fresh fish**								
Yes	151 (86.8)	248 (71.3)	**2.64**	[1.61–4.34]	**0.000**	**4.26**	[1.56–11.65]	**0.005**
No	23 (13.2)	100 (28.7)	1.00 (Ref)			1.00 (Ref)		
**Regular consumption groundnut**								
Yes	148 (85.1)	191 (54.9)	**4.67**	[2.93–7.46]	**0.000**	**6.46**	[2.57–16.27]	**0.000**
No	26 (14.9)	157 (45.1)	1.00 (Ref)			1.00 (Ref)		
**Regular consumption groundnut oil**								
Yes	16 (9.2)	236 (67.8)	**0.04**	[0.02–0.08]	**0.000**	**0.05**	[0.02–0.14]	**0.000**
No	158(90.8)	112 (32.2)	1.00 (Ref)			1.00 (Ref)		
**Regular consumption palm oil**								
Yes	156 (89.7)	229 (65.8)	**4.50**	[2.63–7.69]	**0.000**	2.02	[0.79–5.17]	0.13
No	18 (10.3)	119 (34.2)	1.00 (Ref)			1.00 (Ref)		

L = lower; U = upper; crude OR = crude odds ratio; aOR = adjusted odds ratio; Ref = reference; P = p-value CI = confidence interval; NA = not applicable. OR was calculated by using logistic regression.

^‡^ This parameter was calculated only for women who feed their babies with breast milk.

**Table 4:** Univariate and multivariate analysis of breast cancer, soybean, alcohol, habit of keeping cell phone and money in bras and practice physical activity among women studied.

Factors associated	Cases (174)	Controls (348)	Univariate analysis			Multivariate analysis		
	N (%)	N (%)	crude OR	95%, CI [L-U]	P	aOR	95%, CI [L-U]	P
**Regular consumption soybean**								
**Yes**	152 (87.4)	118 (33.9)	**13.46**	[8.17–22.18]	**0.000**	**16.74**	[7.03–39.84]	**0.000**
**No**	22 (12.6)	230 (66.1)	1.00 (Ref)			1.00 (Ref)		
**Drunk unsweetened coffee**								
**Yes**	1 (0.6)	36 (10.3)	**0.05**	[0.007–0.36]	**0.003**	0.2	[0.02–1.72]	0.14
**No**	173 (99.4)	312 (89.7)	1.00 (Ref)			1.00 (Ref)		
**Consume alcohol**								
**Yes**	154 (88.5)	247 (71.0)	**3.14**	[1.84–5.29]	**0.000**	**2.53**	[1.39–4.60]	**0.002**
**No**	20 (11.5)	101 (29.0)	1.00 (Ref)			1.00 (Ref)		
**Number of alcohol glasses consumed weekly**								
**Never**	16 (9.2)	100 (28.8)	1.00 (Ref)			NA		
**1–9**	154 (88.5)	238 (68.6)	0.36	[0.09–1.30]	0.12			
**≥ 10**	4 (2.3)	9 (2.6)	1.45	[0.44–4.81]	0.53			
**Type of alcohol consumed**								
**Modern beer**	34 (21.5)	155 (62.8)	1.00 (Ref)			1.00 (Ref)		
**Wine**	15 (9.5)	16 (6.5)	**0.15**	[0.09–0.24]	**0.000**	**0.16**	[0.09–0.26]	**0.000**
**Local beer**	109 (69.0)	76 (30.8)	0.65	[0.30–1.40]	0.27	0.7	[0.32–1.55]	0.38
**Habit of keeping cell phone in bras**								
**Yes**	87 (50.0)	240 (69.0)	1.00 (Ref)			1.00 (Ref)		
**No**	87 (50.0)	108 (31.0)	**0.45**	[0.31–0.65]	**0.000**	**0.56**	[0.35–0.89]	**0.01**
**Habit of keeping money in bras**								
**Yes**	122 (70.1)	131 (37.6)	**3.88**	[2.63–5.74]	**0.000**	**3.57**	[2.24–5.69]	**0.000**
**No**	52 (29.9)	217 (62.4)	1.00 (Ref)			1.00 (Ref)		
**Practice physical activity**								
**Yes**	172 (98.2)	315 (90.5)	**0.91**	[0.21–0.98]	**0.03**	**0.71**	[0.14–0.84]	**0.01**
**No**	2 (1.1)	33 (9.5)	1.00 (Ref)			1.00 (Ref)		
**BMI (Kg/m^2^)**								
**< 25**	128 (75.7)	209 (60.8)	1.00 (Ref)			1.00 (Ref)		
**25–29**	40 (23.7)	82 (23.8)	**3.24**	[2.43–23.75]	**0.001**	**5.36**	[4.46–24.57]	**0.000**
**≥ 30**	1 (0.6)	53 (15.4)	**2.58**	[1.45–19.37]	**0.002**	**3.11**	[2.39–20.42]	**0.001**

L = lower; U = upper; crude OR = crude odds ratio; aOR = adjusted odds ratio; Ref = reference; P = p-value CI = confidence interval; BMI = body mass index; NA = not applicable.

OR was calculated by using logistic regression.

### Anthropometric factors and their associations with breast cancer

The weight and height were collected and recorded for all cases and controls included in the study (522 samples). The mean BMI was 24.06 ± 3.11 kg / m^2^ in the cases and 26.29 ± 8.3 kg / m^2^ in controls at the limit of overweight according to WHO criteria. The median was 24.0 kg / m^2^. However, over 71% of cases compared to 62% of controls had a BMI below 25 kg / m^2^. Note that also a small proportion of cases (0.6%) had a BMI above 30 kg / m^2^. Table 5 shows statistically significant relationships between cases and controls regarding height, weight and BMI. Unconditional logistic regression indicated that the odds of having BC were 5.36 and 3.11 times higher in overweight women (BMI = 25–29 kg / m ^2^) and obese (BMI ≥ 30 kg / m ^2^) compared to those with BMI less than 25kg / m^2^ (95% CI, 4.46–24.57; p<0.001 and 95% CI, 2.39–20.42; p = 0.001).

**Table 5:** Distribution of anthropometric variables between cases and controls

Variables	Cases (174)	Controls (348)	Total (522)
	N (%)	N (%)	N (%)
**Height (cm)**			
< 160	57 (32.8)	229 (65.8)	286 (54.8)
160–164	23 (13.2)	14 (4.0)	37 (7.1)
165–170	40 (23.0)	74 (21.3)	114 (21.8)
> 170	54 (31.0)	31 (8.9)	85 (16.3)
**Test for trend**	χ2 = 70.31, **P < 0.001**^a^		
**Mean height (SD)**	165.07 (8.92)	151.47 (18.40)	156.01 (17.12)
**T-test**	t = -9.21, **P < 0.001**^b^		
**Weight (Kg)**			
≤ 50	9 (5.2)	89 (25.6)	98 (18.8)
51–60	47 (27.0)	150 (43.1)	197 (37.7)
61–70	81 (46.6)	72 (20.7)	153 (29.3)
> 70	37 (21.3)	37 (10.6)	74 (14.2)
**Test for trend**	χ2 = 72.84, **P < 0.001**^a^		
**Mean weight (SD)**	65.43 (9.39)	58.14 (10.27)	60.57 (10.55)
**T-test**	t = -7.85, **P < 0.001**^b^		
**BMI (Kg/m2)**			
< 25	124 (71.3)	217 (62.4)	341 (65.3)
25–29	38 (21.8)	76 (21.8)	114 (21.8)
≥ 30	12 (6.9)	55 (15.8)	67 (12.8)
**Test for trend**	χ2 = 9.02, **P = 0.01**^a^		
**Mean BMI (SD)**	24.06 (3.11)	26.29 (8.33)	25.55 (7.11)
**T-test**	t = 3.40, **P = 0.001**^b^		

BMI = body mass index; N = number; % = percent; cm = centimeter; kg = kilogram; χ2 = chi-square; SD = standard deviation; P = *p*-value.

^a^*p*-value was obtained using a Fisher's exact test (two-tailed);

^b^*p*-value was obtained using the independent-sample *t*-test (two-tailed)

## Discussion

The present study shows that education level, marital status, positive family history of cancer, radiation exposure, consumption of: charcuterie, fresh fish, peanut, soybean and alcohol, habit of keeping money in brassiere and high a BMI are risk factors for BC in CAR. Occupation, residence, consumption of wine and groundnut oil, no habit of keeping cell phones in bras, physical activities and unsweetened coffee consumption were protective against BC.

The mean age of BC patients in this study was 45.8 ± 13.5 years, which is slightly below the previous studies in Iran (47.6 ± 10.7 years) and confirms a younger age for BC development among Central African women [20].

According to previous studies, socio-economic status was a strong predictor of health status [21]. Indeed, socio-economic inequalities could affect the diagnostic phase, survival and mortality due to BC patients [22]. Our study found that female employees have a significant protective effect on BC risk compared to housewives. This observation disagrees with the studies in Iran in 2011 and 2015, which focused on the levels of socio-economic status of the family as effective critical risk factors for BC among Iranian women [23,24]. Our results might explain that employed women have generally more family income and can ensure their health insurance. In addition, the economic environment also could affect the willingness of a person to spend money on his / her medical needs. Therefore, we can see better monitoring of health among employed women compared to those who stay at home (housewives).

With regard to education level and its association with BC, some studies have suggested that education level is associated with BC risk [25,26]. However, our study found that BC was more common among less or no educated women than those with a high level of education (university). There is an agreement with the findings from a population-based cohort, 1964–2008 in Israel in 2015 [27], but in contrast to the gradient effect observed in European populations during the 1990s and Norwegian-Swedish women’s lifestyle and health cohort study [19,25]. One explanation for this might be the low number of women with a university education in this study. In addition, African women with a high level of education easily understand the importance of modern medicine compared to those who are uneducated and who believe more in tradition medicine.

Our study found that, coming from an urban environment decreased the risk of developing BC. However, we expected that there would be differences between rural and urban areas because of perceived differences in lifestyle in terms of diet and environmental factors. Our results are in disagreement with cancer epidemiology prevention in 2006 and rural urban differences in BC in India in 2014 that showed a higher risk of BC, respectively, among people living in an urban area and protector for those in the rural area [28,29]. On the other side, the recent study in Uganda in 2016, has suggested no significant association between women in the rural or urban in relation to BC risk [30]. A plausible explanation is in CAR, the only diagnosis laboratory for BC is in Bangui, making access difficult for people living in the provinces. This disadvantage could justify the low prevalence of case among people living in rural areas compared to those living in Bangui.

According to this study, the risk of BC increases among married women compared to unmarried. The difference was statistically significant. Our results were corroborate form India in 2013, who reported that women who had an age of marriage more than 20 years had a 2.69 (95% CI: 1.77–4.07) times higher risk of BC [31]. In contrast, findings reported by the data from the Hong Kong registry in 2016, showed that people who were never married more likely to receive BC surgery [32].

The results of our study showed that positive family histories of BC are highly associated with BC. Especially the mother affected by BC is much more contested than other family members. Our results corroborate some previous studies worldwide which found that family history of BC is a factor associated with BC [33–35].

The International Agency for Research on Cancer (IARC) and the World Cancer Research Fund (WCRF) found that diet strongly influences cancer prevention, and provided advice and recommendations for the management of body weight in BC for primary and secondary prevention [36]. Traditionally, alcohol consumption among women in underdeveloped countries is less common compared to those of developed countries. However, the influences of western culture have exposed African women to a new lifestyle which predisposes them to alcohol abuse, which would be the source of risk in cancer development [9]. According to our results, alcohol consumption was found as a risk factor associated with BC. Our study corroborates recent prospective cohort studies which found alcohol consumption as factors associated with BC [37]. In addition, the Million Women Study (MWS) showed that the relative risk of developing BC increases by 7.1% for each10g / day of alcohol [38]. This relationship between BC and alcohol was in opposition to the study in Bangladesh in 2015 [39]. On the other hand, tobacco consumption, generally associated with risk of BC was found not significant in our study.

Recently, a meta-analysis study published by the National Institute of Public Health (NIPH) in Poland in 2015, showed no significant association between risk of BC and coffee consumption [40], or only a significant beneficial relationship limit between coffee consumption and the high risk of BC [41,42]. Our study found that unsweetened coffee consumption decreases the risk of BC. But after multivariate analysis this association was not significant.

Intensive surveys of several groups showed that the breast is one of the most sensitive organs to radiation effects [43]. The results of our study showed that radiation exposure is highly associated with BC. This finding corroborates the results from the USA, which showed that exposure of breast tissue to ionizing radiation before 40 years old is associated with a three-fold risk of BC for an exhibition valued at 1 Gy [44].

According to our results, groundnut oil consumption significantly reduces the risk of BC. However, consumption of charcuterie, fresh fish, groundnut and soybean was significantly associated with risk of BC. Our results disagree with a study in Mexico City published 2015, who asserted that large groundnut consumption significantly reduces the risk for BC by 2–3 times [45]. Regarding soybean consumption, our results are consistent with those in China from 2015, which have also shown the effect of soy-associated BC increased risk [46]. In contrast, the systematic review and meta-analysis conducted among Chinese females in 2014 showed that soy is a protective food against BC [47]. Regarding the consumption of meat, the Nurses’ Health Study II (NHS II) in the study of 116,671 females registered nurses aged 25 to 43 years in 1989, found no significant relationship between BC and meat [48]. However, another study among Chinese women in 2011 showed that consumption of red meat increases the risk of BC 5.2 times [49]. Our study found no significant relationship between red meat consumption with BC. The findings of the study conducted in China in 2014, found that intake of freshwater fish and their fatty acids may modify risk of BC, and that different species of freshwater fish could have different actions on BC risk. But our study has not specified the different species of fresh fish consumption. This study suggest that future epidemiologic studies are needed to know the effects of different species of fresh fish consumed with BC risk and the cause of these effects [50].

In our study, physical activity was associated with a risk reduction of BC; this confirms the results of prospective studies in the world [51–53]. Conversely, studies conducted in Los Angeles and Western Australia, found the opposite or non-significant effect of physical activity with BC [54,55]. Our results could be explained by the fact that majority of Central African women do not use modern conveniences (car, machines, etc.) for their daily activities.

However, we have found that a non-habit of keeping cell phones in bras significantly reduced BC risk 0.56 times. In contrast, women that used bras to keep money were 3.57 times more likely to achieve BC compared with those who did not have this habit. Indeed, some African women particularly in rural areas have the habit of using bras for a wallet; they completely ignore the fact that it may be a risk factor for BC. In view of the assumptions made, a more specific study will illuminate the effect of the composition of these items compared to BC. Similar studies have not been found to compare our results.

According to the literature review, the higher BMI is significantly increased risk of BC, but with some differences in age and condition of menopause. The association between overweight (defined as a BMI of25 to 29.9kg /m^2^) or obese (BMI ≥ 30 kg /m^2^) and the incidence of BC has been found in numerous studies [46,56]. However, some previous studies showed that no significant correlation was found between weight and BMI with BC risk [23,57]. In addition, the results of our study show that overweight and obesity were associated with BC risk and corroborated a recent study among Iranian females [58].

Some limits must be considered to explain the results of this study. First, the study was carried out on a low population density of CAR; therefore known risk factors may be differing in the general population of women of Central Africa. Another limitation of this study is that the majority of the data was obtained from self-reporting of women. In addition, all information provided to investigators are not entirely reliable, because some questions were using memories such as family history of BC, average alcohol consumed and the duration of a practice session of physical activity. Note also that the quality of data obtained from relatives of deceased cases remains low. However, the results and limitations of the study are very useful in that they contribute to the ongoing research in the field of BC in CAR. In addition, this study was conducted in a developing country where changes in lifestyle can provide other important information about BC risk factors.

## Conclusion

Findings of this study have shown that educational level, marriage, positive family history of cancer, radiation exposure, consumption of charcuterie, groundnut, soybean, alcohol, habit of keeping money in bras, overweight and obesity were associated with BC risk among Central African women in Bangui. Women living in this region should be more cautious on the risk associated with BC. Further studies are needed to investigate other unknown determinants of BC. Finally, a public awareness concerning schooling, occupation, residence area, groundnut oil consumption, physical activities, wine consumption and no habit of keeping cell phone in bras which are protective factors against BC will be useful for the prevention and reduction in the incidence rate.

The results suggest that lack of education and self-awareness in CAR women may be associated with low levels of self-reporting. The results suggest the need for further research to examine other likely behaviors to develop BC in women, and the roles that ethnicity and culture may play in their expression.
